# Successful Case of Cæsarean Section

**Published:** 1829-01-01

**Authors:** 


### LXVIII.

### SANTA MARIA HOSPITAL OF FLORENCE.

SUCCESSFUL CASE OF CÆSAREAN OPERATION. By SIGNIOR TASSINARI.

Eg. Zanobini, 23 years of age, of weak constitution, ricketty, and deformed, entered on the holy state of matrimony, contrary to the advice of her medical and other friends. Six months after marriage, she became pregnant, and on the 11th of May, 1827, being near the completion of the ninth month of utero-gestation, she was seized with labour-pains, but unaccompanied by any expulsive efforts. In 24 hours the waters burst; but the midwife delayed sending for a surgeon, thinking the protraction of delivery was owing to the circumstance of its being a first accouchment. At the expiration of 48 hours, Dr. Lotti was summoned, and found that the superior aperture of the pelvis was too small to permit the descent of the head. The patient was therefore conveyed to the SANTA MARIA HOSPITAL, at 10 o'clock in the forenoon of May 13th, and placed in the ward of St. PHILIPPE. Professor Andrini examined the parts, and came to the conclusion that nothing but the Cæsarian operation offered any chance of success. Several other professors and surgeons came into consultation, and were of the same opinion. The operation being determined on, the head-dresser of the hospital, (le premier elève interne en chirurgie,) M. Tassinari, proceeded to the task.* An incision directly over the linea alba was made, between the umbilicus and pubes, and then the tendinous fibres were divided, till the peritoneum was exposed. This last was opened by means of scissors, and the opening in it enlarged by *tearing the membrane with the fingers*, to the extent of the external incision.† The uterus was next incised, from the fundus to near the cervix. The fœtus was then seized by the feet, and readily extracted. A very slight traction on the umbilical cord served to remove the placenta. The wound was brought together, secured by sutures, and covered with lint. The patient was put to bed, and an anodyne draught exhibited. At six in the evening, there were signs of approaching reaction and inflammation, and twelve ounces of blood were abstracted, which was repeated at midnight. Next morning, (14th of May,) the pulse was very high, and a third venesection was employed. At 2, P. M. vomiting came on, but was stopped by the exhibition of iced drink. At 11 o'clock, the abdomen was much swelled, and the fever very high, with sanguineous discharges from the vagina, and suppression of urine. Bled again, to six ounces—catheter introduced, but no water drawn off—laxative enema. 15th. The constipation and ischuria continue—fever intense—enemata—another venesection. 16th. The symptoms are improved—the fever reduced—the abdomen less tense—the lochia flowing copiously. The urine has again appeared—and the patient has slept. 17th. An aggravation of the general and local symptoms—another venesection. The symptoms now became more favourable. On the 21st, the dressings were removed, in the presence of all the professors who had assisted at the operation, and the whole line of incision was found to be united, except a very small portion at the inferior angle, where a piece of lint had been introduced between the lips. On the 7th of June, the patient was able to get out of bed, and was discharged from the hospital cured on the 16th of the same month.

The foregoing operation, authenticated as to its success, beyond the slightest doubt or cavil, does great credit to the juvenile operator. It appears that this was the first successful operation of the kind, which was performed in the hospital above-mentioned.

---

* This is a plan adopted in several foreign schools; but which, we suppose, John Bull would not permit. In this country, students have few opportunities of putting the knife upon the living body, till, in private practice, they have the whole responsibility on their heads, and none to direct their trembling hands! The continental rule of permitting students to operate under the direction of their masters, is assuredly better calculated for sending forth into private life men capable of operating, than the plans here prevailing.

† The operator gives no reason for this tearing of the peritoneum, instead of making a clean incision with the bistoury—nor can we conceive any good reason why the above process was employed. The issue was successful; but this does not justify, in our opinion, the measure pursued.

